# MoS_2_-Based nanocomposite for synergistic chemo-photothermal cancer therapy

**DOI:** 10.1038/s41598-025-05615-8

**Published:** 2025-07-02

**Authors:** Maryam Mahmoodabadi, Mohammad Taghi Goodarzi, Nasrin Salehi, Alireza Jalali, Ehsan Zahedi

**Affiliations:** 1 Department of Biochemistry, Shahrood Branch, Islamic Azad University, Shahrood, Iran; 2 Department of Basic Sciences, Shahrood Branch, Islamic Azad University, Shahrood, Iran; 3 Department of Chemistry, Herbal Medicines Raw Materials Research Center, Shahrood Branch, Islamic Azad University, Shahrood, Iran

**Keywords:** MoS_2_ nanoflakes, Nanocomposite, DOX delivery, Photothermal therapy, Biotechnology, Nanoscience and technology

## Abstract

MoS₂ nanoflakes are emerging as a promising material for photothermal therapy due to their high absorption in the NIR region, large specific surface area, biocompatibility, efficient photothermal conversion, and ability to be functionalized for targeted therapy. In this paper, MoS₂ nanoflakes were incorporated to Fe_3_O_4_ nanoparticles, gold nanorod (GNR), and copper sulfide (CuS) (MCG nanocomposite) to investigate chemo-photothermal therapy in this nanocomposite. The structural and optical properties of the MCG nanocomposite were characterized by X-ray diffraction (XRD), Transmission electron microscopy (TEM), Zeta potential, Dynamic Light Scattering (DLS), Fourier transform infrared (FTIR), and Ultraviolet-visible (UV-Vis) spectroscopies. The photothermal results of samples indicated that MCG nanocomposite produced higher photothermal heat than each individual sample alone (808 nm NIR laser irradiation at a power density of 1 W/cm^2^ after 10 min). Under NIR laser irradiation, the release of DOX was greatly accelerated at pH = 5.5 as compared to pH = 7.4. So, this nanocomposite can be used as dual responsive systems, with DOX release controllable through pH and NIR irradiation. Finally, MTT assays experiment showed that, using NIR irradiation, the relative viabilities of HeLa cells decreased when the concentration of drug increased. Hence MCG nanocomposite could be a potent system for targeted drug delivery and synergistic chemo-photothermal cancer therapy.

## Introduction

Photothermal refers to the process where light (typically infrared or visible) is absorbed by a material and converted into heat^1^. This produced heat is used in various applications including: solar energy harvesting^2^, medical treatments^3^, and in materials science^4,5^. Photothermal therapy is a newly developed therapeutic strategy, which employs the near-infrared (NIR) laser photoabsorbers to generate heat for thermal ablation of cancer cells upon NIR laser irradiation. To date, a variety of photothermal agents including gold nanoparticles^6,7^, copper nanoparticles (CuS)^8,9^, carbon nanomaterials^10^, transition metal chalcogenides^11,12^, silver nanostructure^13^, etc., have been extensively explored to produce heat up and destroy the cancer cells.

Molybdenum disulfide (MoS₂) is one of the most stable layer transitional metal dichalcogenides which each layer is consisted of a plane of molybdenum atoms sandwiched between two layers of sulfur atoms^11,14^. These layers are held together by weak Van der Waals forces, allowing the material to exhibit unique properties like high lubrication^15^, electrical conductivity^16^, drug delivery^17^, photothermal therapy^4,11^, and semiconducting behavior^18^.

MoS₂ nanoflakes possess high surface area which can enhance their interaction with biological molecules and improve drug delivery and targeting capabilities in these nanoflakes. This large surface area also is applied for easy functionalization, where targeting ligands (such as antibodies, peptides, or small molecules) can be attached to direct MoS₂ nanoparticles to specific cancer cells, minimizing off-target effects^19,20^. MoS₂ nanoflakes also exhibit strong NIR absorption, making them ideal candidates for photothermal applications. Their ability to absorb light in the NIR region allows them to penetrate deeper into tissues, enabling effective treatment of tumors that are located in deeper tissues. Also, MoS₂ nanoflakes demonstrate excellent photothermal conversion efficiency, biocompatibility, and low toxicity^21^. Due to mentioned properties, MoS₂ nanoflakes can be used as carriers for loading therapeutic drugs. When functionalized with targeting molecules, they could deliver drugs directly to the tumor site and then be activated by near-infrared (NIR) light for enhanced therapeutic effects^11^.

Gold nanorods (GNRs) are a type of material with a rod-like shape that exhibit unique optical properties, especially when they interact with light^22^. Due to their size, tunable optical properties, and biocompatibility, these nanorods are often used in sensors, imaging, and drug delivery systems^23–25^. They also show a strong absorption in the NIR region, making them a photothermal agent at converting light into heat. This property makes them particularly useful in medical therapies like photothermal therapy, where they can be used to target and destroy cancer cells without affecting surrounding healthy tissue^26^.

CuS nanoparticles are another type of nanomaterial that have attracted the most attentions in photothermal therapy due to strong absorption in the NIR region, low toxicity, cost effectiveness, thermal conversion efficiency^9,27^. In addition to photothermal therapy, CuS nanoparticles can also be applied in other therapeutic modalities such as drug delivery, and photoacoustic imaging^28^.

Iron (III) oxide (Fe₃O₄), commonly known as a superparamagnetic nanoparticle with unique properties that make it highly effective for smart drug delivery systems. Due to its biocompatibility, magnetic properties, and ability to functionalize for targeted therapy, Fe₃O₄ nanoparticles have been extensively studied in the field of nanomedicine. These nanoparticles are magnetically responsive, meaning they can be controlled by an external magnetic field. This property allows precise targeting of drugs to specific areas in the body, such as tumors or inflamed tissues, by applying a magnetic field. This magnetic targeting ensures that drugs are delivered directly to the affected site, increasing therapeutic efficacy and reducing side effects^29,30^.

Recently, nanotechnology has combined methods for fast therapy using multifunctional nanocomposites based on nanoparticles and layer nanostructures to quickly destroy cancer cells. In this study, our aim is to achieve the improvement magnetic targeting photothermal therapy of MoS_2_ nanoflakes. To do this, CuS nanoparticles, GNRs were incorporated in MoS_2_ nanoflakes to obtain MoS_2_/CuS/GNR (MCG nanocomposite). After that, Fe₃O₄ nanoparticles were used for the targeted treatment of solid tumors. When using these materials, a magnet is used to create an external magnetic field, therefore these nanoparticles will be more concentrated in the tumor area and healthy cells have the least contact with them. Then, MCG nanocomposite was coated with hydrophilic poly ethylene glycol (PEG) polymer to increase the colloidal dispersity and stability of synthesized nanocomposite in aqueous media. Finally, a potent anticancer doxorubicin (DOX) was chosen for delivery by MCG nanocomposite, and the release of drug was evaluated by irradiation of NIR light. The results of current search show that MCG nanocomposite could be used as a smart controllable drug delivery system by photothermal effect.

## Experimental details

### Materials

Copper (II) chloride dehydrate (CuCl_2_·2H_2_O, ≥ 99.0%), thioglycolic acid, and thioacetamide ((CH_3_CSNH_2_, ≥ 99.0%) were purchased from Sigma Aldrich. Ammonium molybdate tetrahydrate ((NH_4_)_6_Mo_7_O_24_·4H_2_O, ≥ 99.0%), polyethylene glycol 400 (H(OCH_2_CH_2_)_n_OH, PEG 400), hydrazine hydrate (50%, v/v), ferric trichloride anhydrous(FeCl_3_), ascorbic acid, hexadecyltrimethylammonium bromide (CH_3_(CH_2_)_15_N(Br) (CH_3_)_3_, CTAB, ≥ 99%), hydrogen tetrachloroaurate (HAuCl_4_.3H_2_O, ≥ 99.9%), sodium borohydride (NaBH_4_, ≥ 98%), hydroquinone (C_6_H_4_(OH)_2_, 99%), and silver nitrate (AgNO_3_, ≥ 99.0%) were purchased from Merck company. All these materials were used without any further purification.

### Synthesis of MCG nanocomposite

The synthesis of the MCG nanocomposite involves four main steps.

I. **Synthesis of MoS**_**2**_**/Fe₃O₄ Nanoflakes**: MoS₂/Fe₃O₄ nanosheets were prepared using a two-step hydrothermal method^18^.
II. **Synthesis of GNRs**: GNRs were synthesized via the seed-mediated method^31^. In this method, in the first step, the spherical gold seed solution was prepared as follows: 364.4 mg of CTAB aqueous solution (5 mL) was added to 5 ml of 0.5 mM hydrogen tetrachloroaurate aqueous solution under stirring. After that, 600 µL of 10 mM sodium borohydride aqueous solution was added to the above mixture, it was shown that the solution’s color immediately changed from yellow to light brown. In GNR growth step, 182.2 mg of CTAB was mixed to 22 mg hydroquinone in 5 mL de-ionized water under ultrasonication. After that, 200 µl of 4 mM silver nitrate aqueous solution, and 5 ml of 1 mM hydrogen tetrachloroaurate aqueous solution were added to the above solution. Finally, 12 µL of the spherical gold seeds suspension was added to gained solution. To complete the GNRs growth process, suspension was kept undisturbed for 24 h before purification at 27 °C. The GNR suspension was centrifuged twice (6000 rpm, 30 min) to remove excess CTAB, and then dispersed in de-ionized water.

I. **Synthesis of CuS Nanoparticles**: First, 200 mL of CuCl₂0.2 H₂O aqueous solution (0.1 mmol) was added to 28.4 µL of thioglycolic acid aqueous solution (0.2 mmol) under constant stirring. Next, a 1 M NaOH aqueous solution was added drop-wise to the mixture while adjusting the pH to 9.0. Then, 40 mL of thioacetamide aqueous solution (0.1 mmol) was added into gained solution. Finally, the solution was heated at 50 °C for 2 h to promote nanoparticle growth.
II. **Formation of the MCG Nanocomposite**: To assemble the MCG nanocomposite, 0.05 g of the synthesized MoS₂/Fe₃O₄ powder was dispersed in 20 mL of deionized water. Finally, 5 mL of the GNR and CuS solutions were separately added drop-wise to the MoS₂/Fe₃O₄ solution under continuous stirring.

### Cell experiments

In this study, HeLa cells were used to investigate cell viability. The cells were cultured in normal DMEM medium supplemented with 10% fetal bovine serum phosphate-buffered saline (PBS). Cytotoxicity was then assessed in vitro using the standard Cell Counting Kit-8 (CCK-8) assay.

#### DOX loading into MCG - PEG

PEG is a hydrophilic and biocompatible polymer that enhances the physiological stability of samples in the body. In this study, MoS_2_ and the MCG nanocomposite were coated with PEG.

For DOX loading into the MCG nanocomposite, 2 mg of MCG sample was first dispersed in 2 mL of an aqueous DOX solution (mg/mL). The mixture was then stirred overnight in a dark room. Finally, the DOX-loaded nanocomposite was centrifuged and washed until the supernatant became colorless, indicating the removal of unbound DOX.

#### DOX release under NIR irradiation

To evaluate DOX release under NIR irradiation, 0.1 µg/mL of DOX-MCG-PEG was incubated in PBS at two different pH levels (pH = 5.5, and 7.4). The samples were then exposed to NIR irradiation (1 W/cm²), and at predetermined time points, the fluorescence of DOX was measured to assess its release.

#### Cell viability analysis

In the first part of this experiment, we assessed the cytotoxic effect of NIR laser irradiation on cells without using DOX. HeLa cells were seeded into a 96-well culture plate and incubated for 24 h. The cells were then exposed to NIR irradiation (1 W/cm²) for a predetermined duration and incubated overnight. For experiments involving DOX and DOX-loaded nanocomposites, the cell culture medium was replaced with fresh medium containing a predetermined concentration of DOX, followed by overnight incubation. Finally, cell cytotoxicity was evaluated using the MTT assay.

Using spectrometer at wavelength of 495 nm, the drug loading capacity and entrapment efficiency were calculated the formulas (1) and (2), respectively:

1 $$\text{Loading Capacity}\: (\text{LC}\:{\%}) = [(\text{m}_0-\text{m}_1)/\text{m}_2] \times 100$$

2 $$\text{Entrapment Efficiency}\: (\text{EE} {\%}) = [(\text{m}_0-\text{m}_1)/\text{m}_0] \times 10$$

Where m_0_ is initial mass of DOX before loading, m_1_ is supernatant free amount of DOX after loading, and m_2_ is nanocomposite mass.

#### Characterization of the samples

Powder X-ray diffraction (XRD) patterns were obtained using an X-ray diffractometer STADI STOE with CuKα irradiation (λ = 1.5405 Å) over a 2θ range of 10–80°. Data were analyzed using X’Pert software. Transmission electron microscopy (TEM) analysis was performed using a Tecnai G^2^ F20 (200 kV scanning transmission electron microscopes). The zeta potential and dynamic light scatting (DLS) analysis of MCG nanocomposite was performed on a Nano–flex 2. UV-Vis spectroscopy was conducted using a Perkin Elmer LAMBDA 20 UV-Vis spectrometer. Fourier-transform infrared (FTIR) spectroscopy was conducted using a Bomem Fourier-transform infrared spectrometry with a resolution of 4 cm⁻¹ over 64 scans. The photothermal effect was measured by using a diode laser (Fiber Coupled Diode Laser, 808 nm, 10 w) when was irradiated perpendicular to a quartz cell containing aqueous solution of samples (total volume = 2 mL). HeLa cell line was purchased from the cell bank of Mashhad University of Medical Sciences of Iran.

## Results and discussions

### Characterization of nanocomposite

**Fig. 1:**
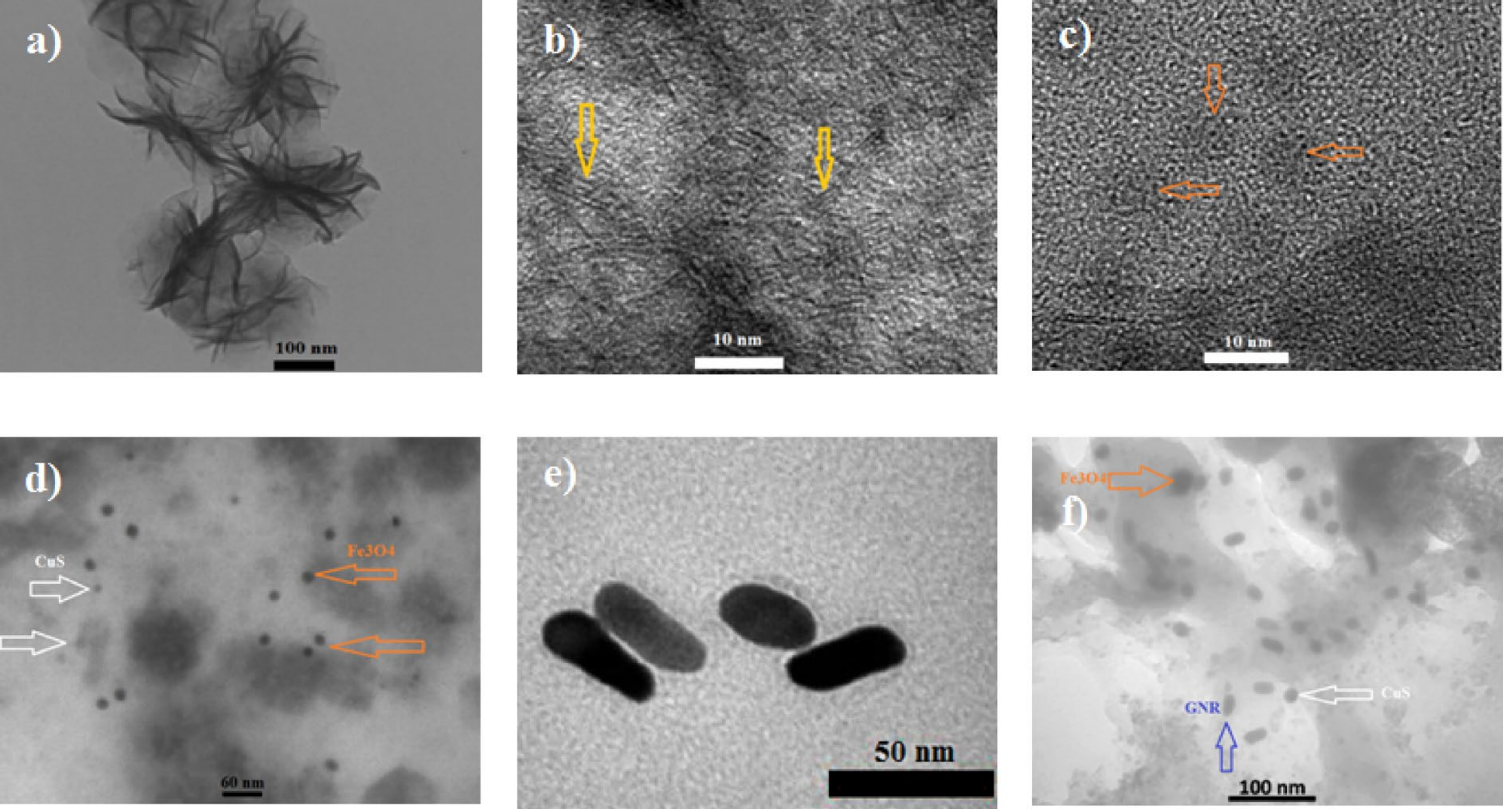
TEM images of **(a, b)** MoS_2_ nanoflakes, **(c)** MoS₂/Fe_3_O_4_, **(d)** MoS₂/Fe_3_O_4_/CuS, **(e)** GNRs, and **(f)** MCG nanocomposite in different scales.

Figure 1(a-f) presents TEM images of MoS₂, MoS₂/Fe_3_O_4_, CuS, GNRs, and the MCG nanocomposite. Figure 1(a, b) reveals that MoS₂ adopts a flower-like structure, consisting of numerous interlaced nanoflakes with diameter of about 180–300 nm and an average thickness of about 2 nm (3–6 layers). In Fig. 1c, Fe_3_O_4_ samples with diameter at about 25 nm randomly distributed over the MoS_2_ nanoflakes. Figure 1d exhibits the CuS material with nanospherical morphology and diameters of approximately 11 nm which were incorporated in between MoS_2_/Fe_3_O_4_ sample. Figure 1e indicates the GNRs with an aspect ratios (length divided by width) of 2.4 synthesized by seed-mediated method. Therefore, the longitudinal plasmon peak of GNRs with such average aspect ratio is estimated at about 700–800 nm wavelength which agree with other reports^31^.

As shown in Fig. 1f, the MCG nancomposite also retains a flower-like morphology; however, this structure results from the decoration of Fe_3_O_4_ nanoparticles, CuS nanoparticles, GNRs by MoS₂ interlaced nanoflakets, which are approximately 5 nm thickness and 180–300 nm diameter. Also, any significant change was not observed in size of these nanoparticles after that were imbedded between MoS_2_ nanoflakes.

**Fig. 2:**
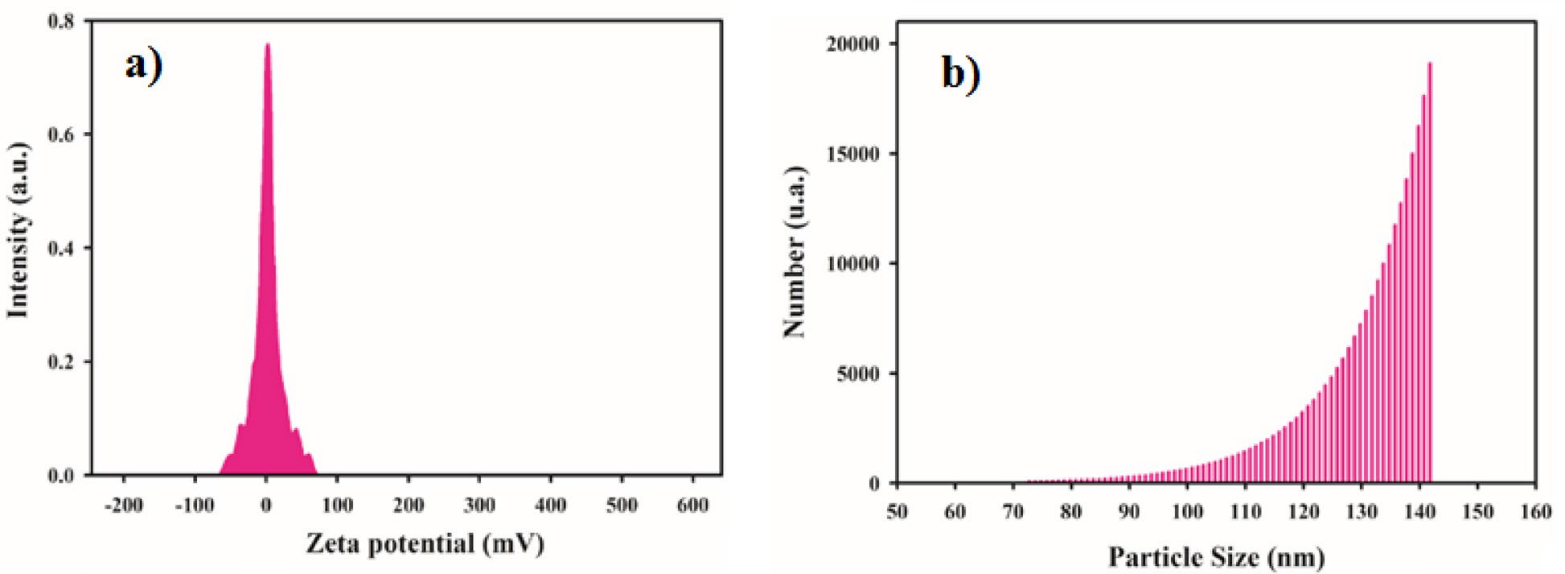
**(a)** Zeta potential diagram, and **(b)** DLS results for MCG nanocomposite.

Figure 2a shows the zeta potential diagrams of MCG nanocomposite measured at 25 °C. The zeta potential was + 2.73 mV, which actually determines the surface charge of nanocomposite. The MoS2 nanoflakes exhibited a zeta potential of −38.2 mV, which changed to + 2.73 mV when decorated with Fe_3_O_4_ nanoparticles, CuS nanoparticles, GNRs. The zeta potential changed from negative to positive due of the presence of amino groups in PEG.

To assess the lateral size distribution of MCG nanocomposite, DLS analysis was conducted. Figure 2b presents the number as a function of particle size for a MCG nanocomposite dispersion sample. The lateral size of the nanocomposite ranges from approximately 50 to 142 nm which is appropriate for use as cell-targeted drug delivery system.

**Fig. 3:**
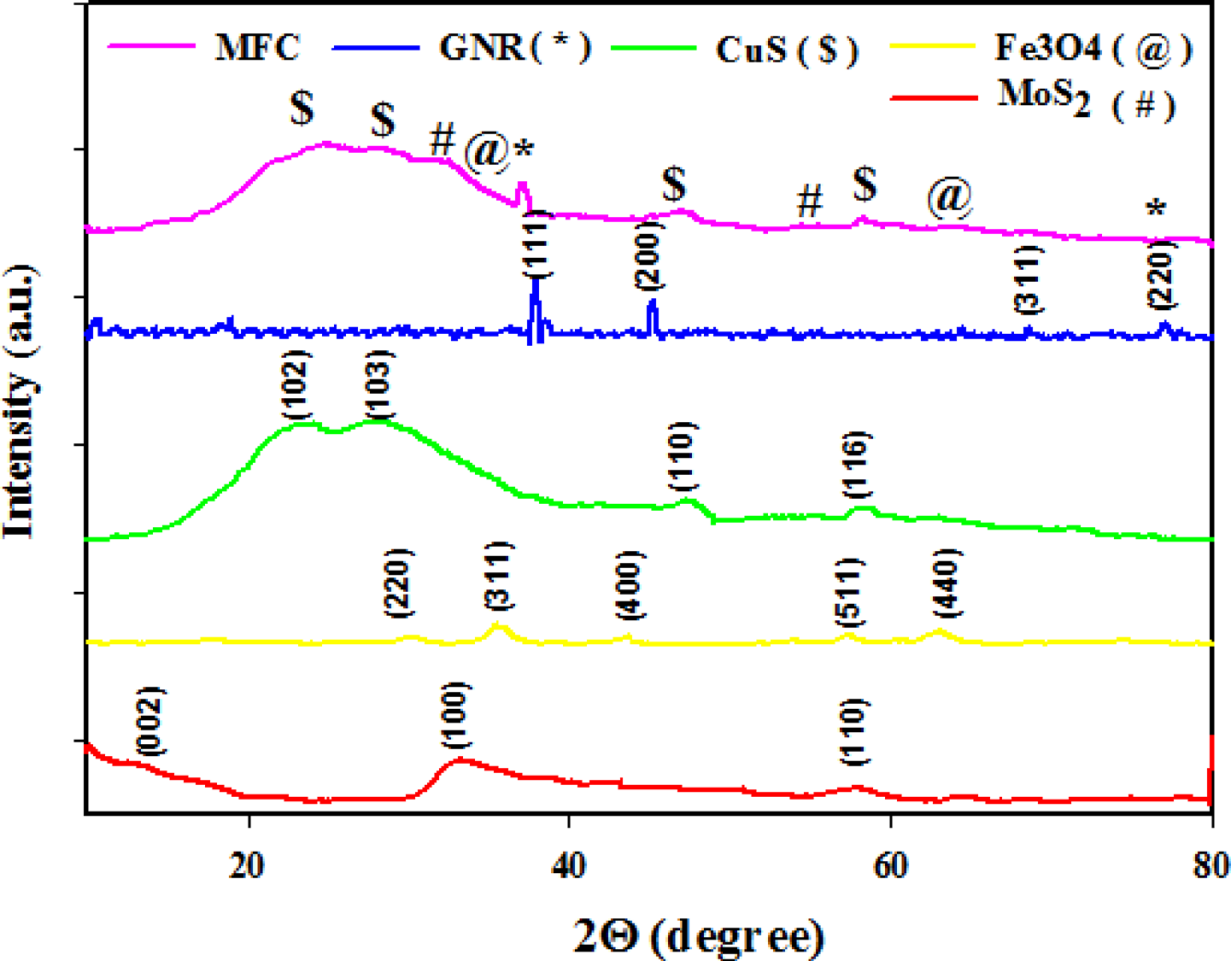
XRD pattern of MoS₂, Fe₃O₄, CuS, GNRs, and MCG nanocomposite.

The phase structure of the obtained samples was examined using XRD analysis. The XRD patterns of MoS₂ nanoflakes (red line), Fe₃O₄ nanoparticles (yellow line), CuS nanoparticles (green line), GNRs (blue line), and the MCG nanocomposite (pink line) are presented in Fig. 3. As shown in the figure, the diffraction peaks are indexed accordingly for all samples. In red line, peaks 13.9°, 33.2°, and 59.4° correspond to the (0 0 2), (1 0 0), and (1 1 0) crystal planes respectively, indicate a hexagonal structure with a space group of P6₃/mmc for MoS₂ nanoflakes (JCPDS Card No. 00–006-0097)^32^. In yellow line, peaks 30.1°, 35.5°, 43.1, 57.1, and 62.6° correspond to the (2 2 0), (3 1 1), (4 0 0), (5 1 1), and (4 4 0) cubic phase of Fe₃O₄ crystal planes, respectively (JCPDS Card No. 01–075-0033)^33^. In green line peaks at 28.9°, 31.7°, 48.4°, and 58.3° correspond to reflections from the (1 0 2), (1 0 3), (1 1 0), and (1 1 6) crystal planes respectively, indicate a cubic CuS structure (JCPDS Card No. 01–006-0464)^34^. In blue line, located peaks at 36.9°, 45.2°, 68.9° and 77.1° angles correspond to the (1 1 1), (2 0 0), (2 2 0) and (3 1 1) planes, indicate a face centered cubic structure with fd3m space group for GNRs (JCPDS Card No 008–0234)^35^. As seen in the pink line, all characteristic peaks of the individual components are present in the XRD pattern of the MCG nanocomposite. This confirms that Fe₃O₄, CuS nanoparticles, and Au NRs are embedded within the MoS₂ nanoflakes.

**Fig. 4:**
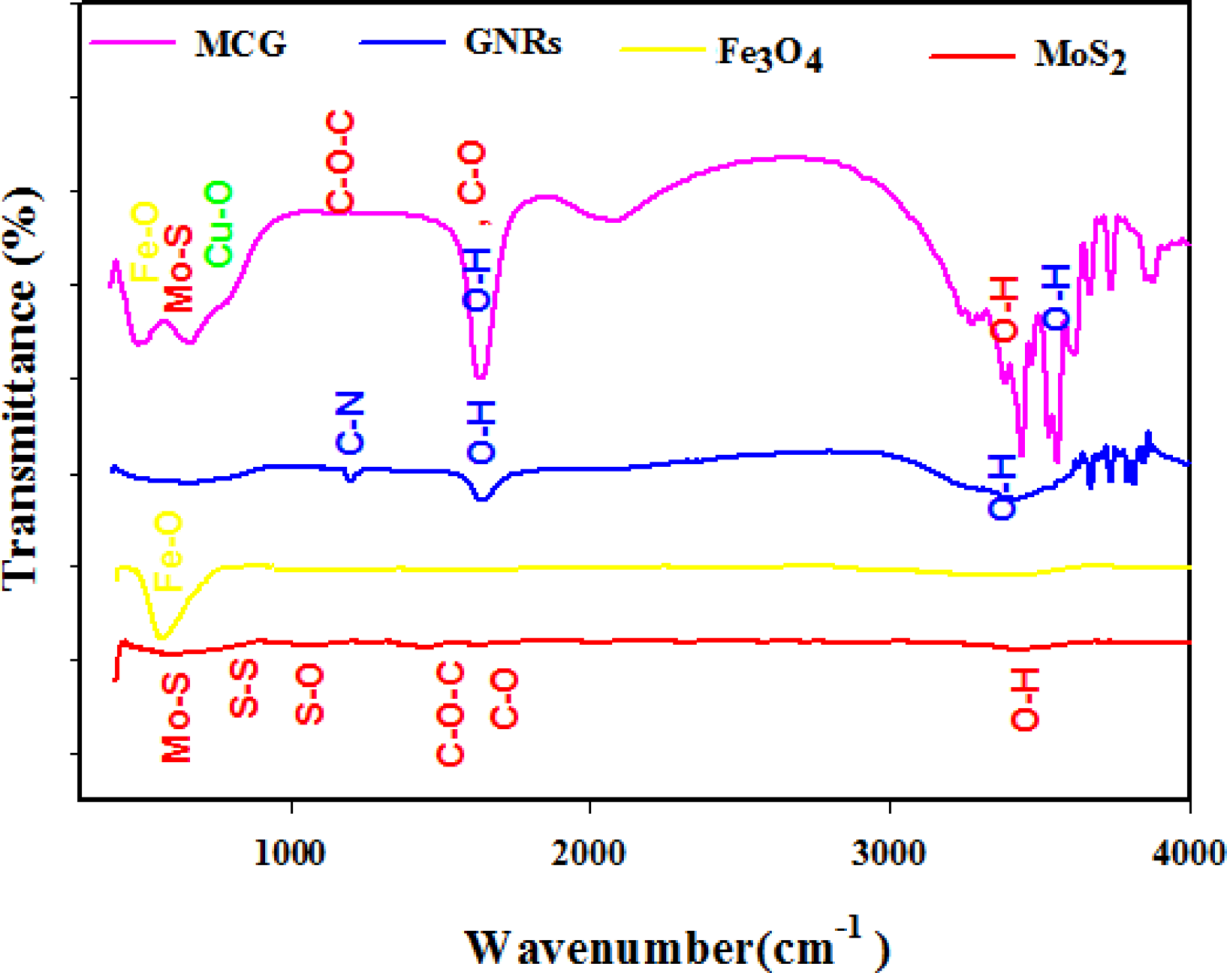
FTIR spectroscopy for MoS_2_, Fe₃O₄, GNRs, and MCG nanocomposite.

To elucidate chemical composition and bond identification present in product molecules, FTIR technique was used. Figure 4 shows the FTIR spectrum of the prepared MoS_2_ nanoflakes, MoS_2_/Fe₃O₄, GNRs, and MCG nanocomposite. Broad absorption bands at 596 cm^−1^, 720 cm^−1^, 1051 cm^−1^, 1438 cm^−1^, 1630 cm^−1^, and 3432 cm^−1^ wavenumber were attributed to MoS_2_ nanoflakes. The peak at about 596 cm^−1^ wavenumber corresponds to Mo–S bond vibration. The peaks at about 720 cm^−1^ and 1051 cm^−1^ are due to S–S and S–O bonds stretching, respectively. The peaks at about 1438 cm^−1^ and 1630 cm^−1^ are ascribed C-O-C, C-O bond stretching vibration adsorbed moisture during synthesis process. Also, broad peak 3432 cm^−1^ represents formation of symmetrical stretching vibration of O–H in PEG^32,35^. This characterization confirms that PEG has modified the surface of MoS_2_. In FTIR spectrum of Fe₃O₄ (yellow line), one peak at about 570 cm^−1^ is indexed as Fe–O bond vibrations^29^. In blue line, a weak bond at 1200 cm^−1^ attributed to C-N stretching of CTAB. In FTIR spectrum of GNRs also indicate a strong broad peak in 3455 cm^−1^ and a weak peak in 1640 cm^−1^, assigned to O-H stretching of the adsorbed H_2_O^36^^,^^37^. The peaks at about 692 cm^−1^ signify the existence of Cu-O bond^20,34^. All of mentioned peak exist in FTIR spectrum of MCG, so the successful synthesis of MCG nanocomposite was confirmed by FT-IR spectroscopy.

**Fig. 5:**
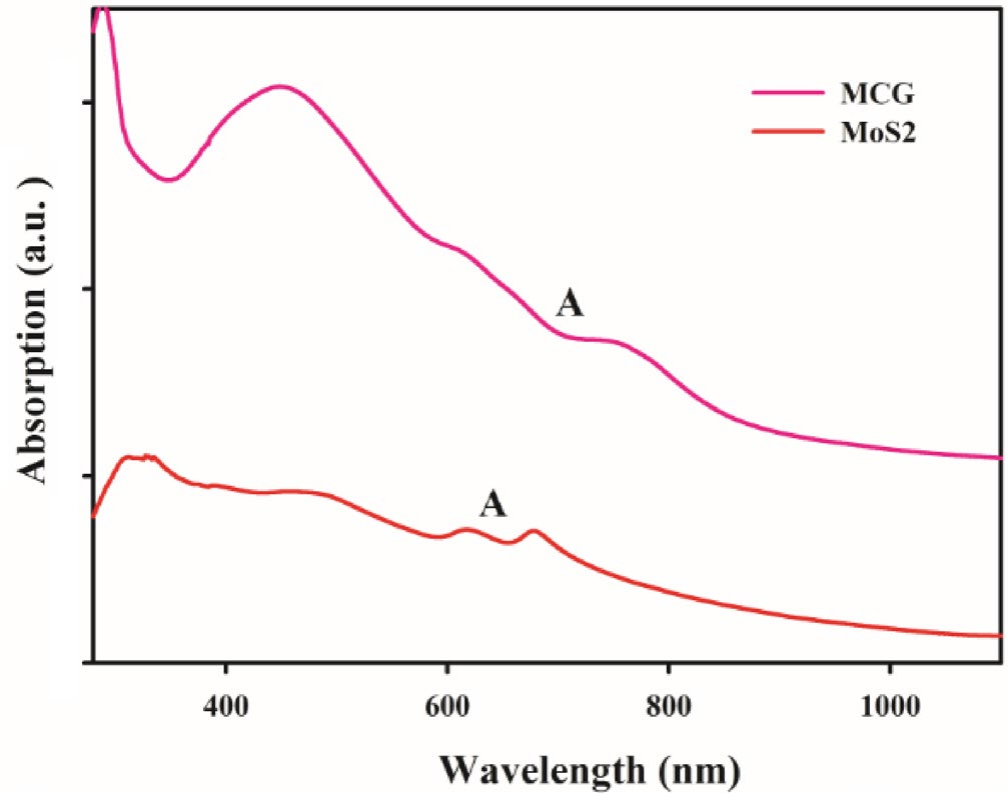
UV-Vis spectroscopy of MoS_2_ nanoflakes (red line), and MCG nanocomposite (pink line).

Figure 5 exhibits UV–Visible spectrum for MoS_2_ nanoflakes (red line), and MCG nanocomposite (pink line) to characterize their optical property. As reported in other literatures, excitonic peaks at *A* = 672 nm wavelength determines the band gap (E = 1360/λ (nm)) in these nanoflakes^11^^,^^38^. On the other hands, band gap can estimate the number (thickness) of layers in nanoflakes that it is between 3 and 6 layers in our synthesized sample which is in agreement with TEM images. As seen in this figure, the amount of absorption spectrum of MCG nanocomposite (pink line) is higher than pure MoS_2_ nanoflakes in all of wavelengths (with the same concentration). Also, this figure confirms that the number of layers (thickness) in nanocomposite is more than pure MoS_2_, because the position of *A* excitonic peak (λ = 760 nm) is bigger than pure MoS_2_ nanoflakes (680 nm).

### Photothermal activity

**Fig. 6:**
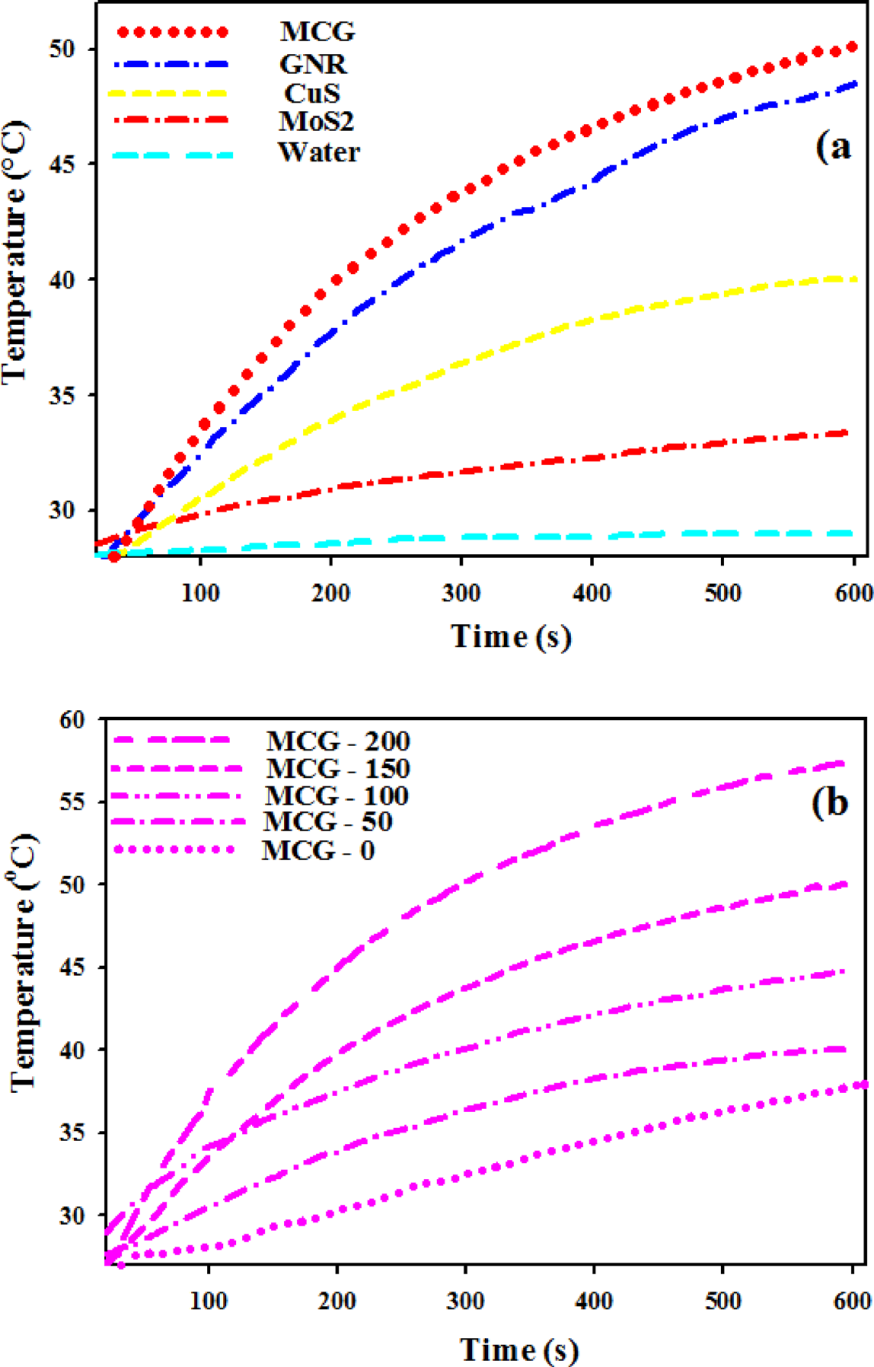
Plot of temperature versus time for **(a)** water, MoS_2_, CuS, GNRs, and MCG solutions, **(b)** MCG nanocomposite with different concentrations (0 to 200 mg/mL) under 808 nm laser irradiation (1 W/cm^2^) for 10 min.

Given the advantages of NIR region in biomedical studies, such as deep penetration of irradiation to tissues, the NIR region is a favorable irradiation for biomedical studies. Thus, an 808 nm laser irradiation at a power density of 1 W/cm^2^ was applied to study the photothermal effect of the MoS_2_, CuS, GNRs, and MCG samples by thermocouple thermometer. As shown in Fig. 6a, for the control experiments, the temperature of de-ionized water revealed no significant increase under NIR laser irradiation after 10 min. As seen in this figure, the temperature of the MoS_2_ solution (150 mg/mL) increased from 28.0 to 33.3 ◦C while the temperature of CuS, GNR, and MCG solutions showed a rapid increase from 28.0 °C to 40.0 °C, 48.4 °C, and 50.0 °C, respectively (in the same concentrations).

As reported in previous literatures^3,9^, the strong absorption of GNRs in the NIR region compared with MoS_2_ and CuS, ensures that its photothermal efficiency would be dramatically enhanced when a laser is used to irradiate the nanomaterial. Figure 5 also shows that developed MCG nanocomposite has a higher absorption compared to pure MoS_2_. This strong absorption of the nanocomposite in this region motivated us to explore its photothermal properties. The photothermal effect of MCG nanocomposites was investigated by measuring the temperature changes in nanocomposite solution at various concentrations (0, 50, 100, 150, 200 $\:\mu\:g/mL$) under 808 nm laser irradiation. As shown in Fig. 6b, with an increase in the concentration of nanocomposites, the temperature of the solutions increased rapidly. Furthermore, the temperature of the solution with 200 $\:\mu\:$g/mL concentration showed a 19 °C increase (from 28 °C to 57.0 °C) after 808 nm laser irradiation for 10 min, while the pure water showed negligible changes. The final temperatures of the nanocomposites with other concentrations (0, 50, 100, 150$\:\:\mu\:g/mL$) were 37.9 °C, 40.1 °C, 44.8 °C, and 50.0 °C respectively, under laser illumination after 10 min. These data indicate that this nanocomposite could act as an efficient photothermal agent.

For photothermal therapy, a minimum temperature of 50 °C was able to destroy cancer cells^39^. Therefore, an irradiation time of 1 min was set for MCG nanocomposite with concentration of 200 $\:\frac{\mu\:g}{mL}$ in the application of photothermal therapy.

### In vitro drug release

**Fig. 7:**
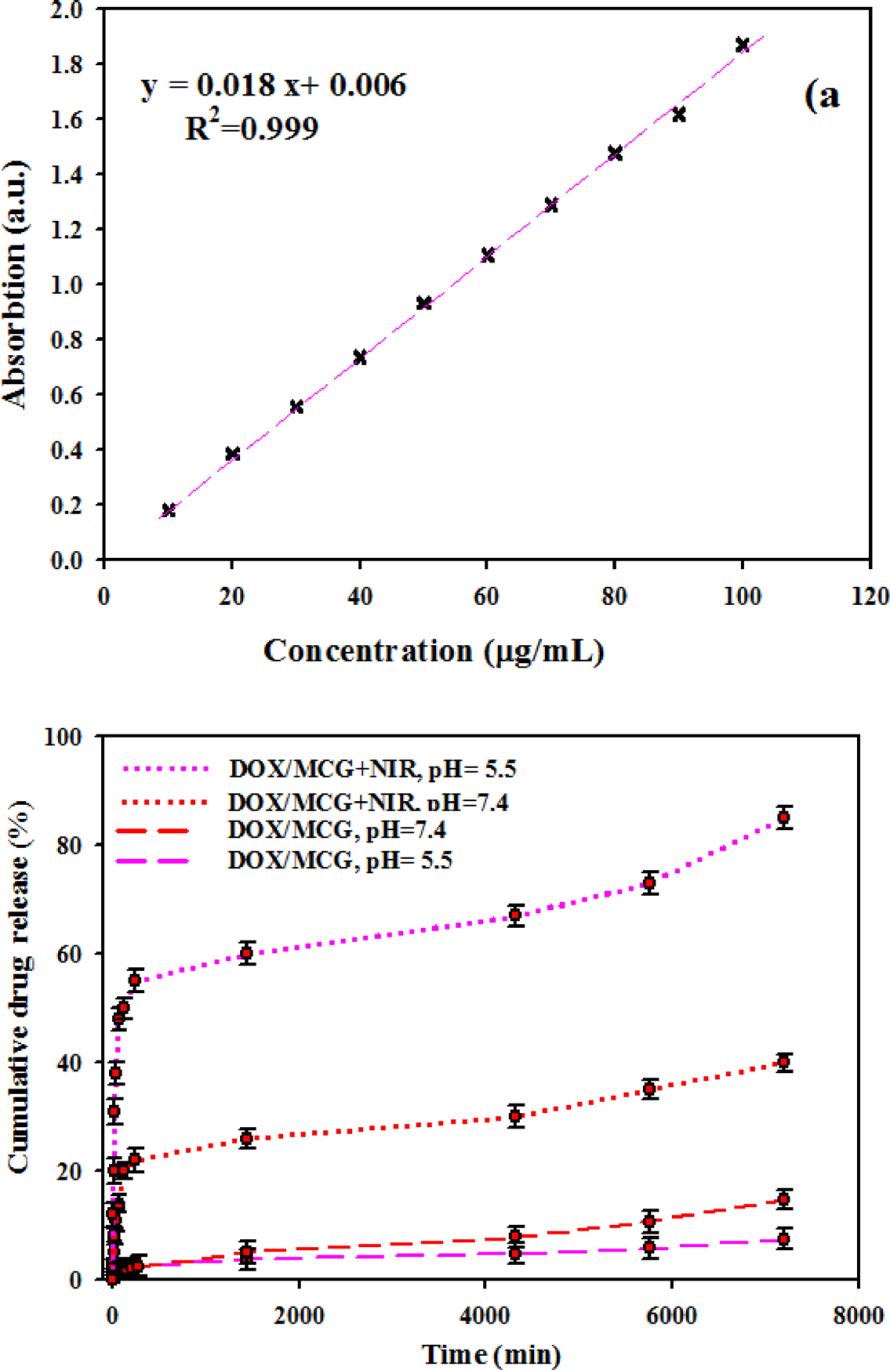
**(a)** Calibration graph of absorbance versus concentration for DOX-MCG, **(b)** Cumulative release of DOX versus time from MCG with, and without 808 nm laser irradiation.

To evaluate the potential of using DOX/MCG/PEG as a drug delivery system, controllable drug release behavior of the nanocarrier was investigated. Figure 7a shows absorption spectroscopy versus drug concentration in MCG nanocarrier. As seen in this figure, there is a linear relationship between the absorbance value and the concentration. According to the Lambert Beer Law, there is a linear increase of absorbance with concentration at a particular wavelength. Using Eq. (1), the loading capacity of DOX in nanocomposite was calculated 19.8%.

The in vitro release behavior of DOX was investigated in two different pH media (5.5 and 7.4) with and without laser irradiation (Fig. 7b). At pH = 5.5, the released DOX from the DOX/MCG-PEG was less than 8% without laser irradiation after 2 h. In contrast, DOX release exceeded 84% after 2 h laser irradiation, which was higher than that without irradiation. Also, in the presence of laser irradiation, the cumulative release of DOX after 2 h at pH = 5.5 (84%) was much greater than that at pH 7.4. These results indicate that the NIR light-triggered photothermal heating effect leads to the vibration of the MoS_2_ nanoflakes and consequently decreases the interaction between DOX and the nanocarrier which could promote the release of DOX. Thus MCG nanocomposite can be beneficial in drug delivery and photothermal therapy^40^.

### Cell vibiability

**Fig. 8:**
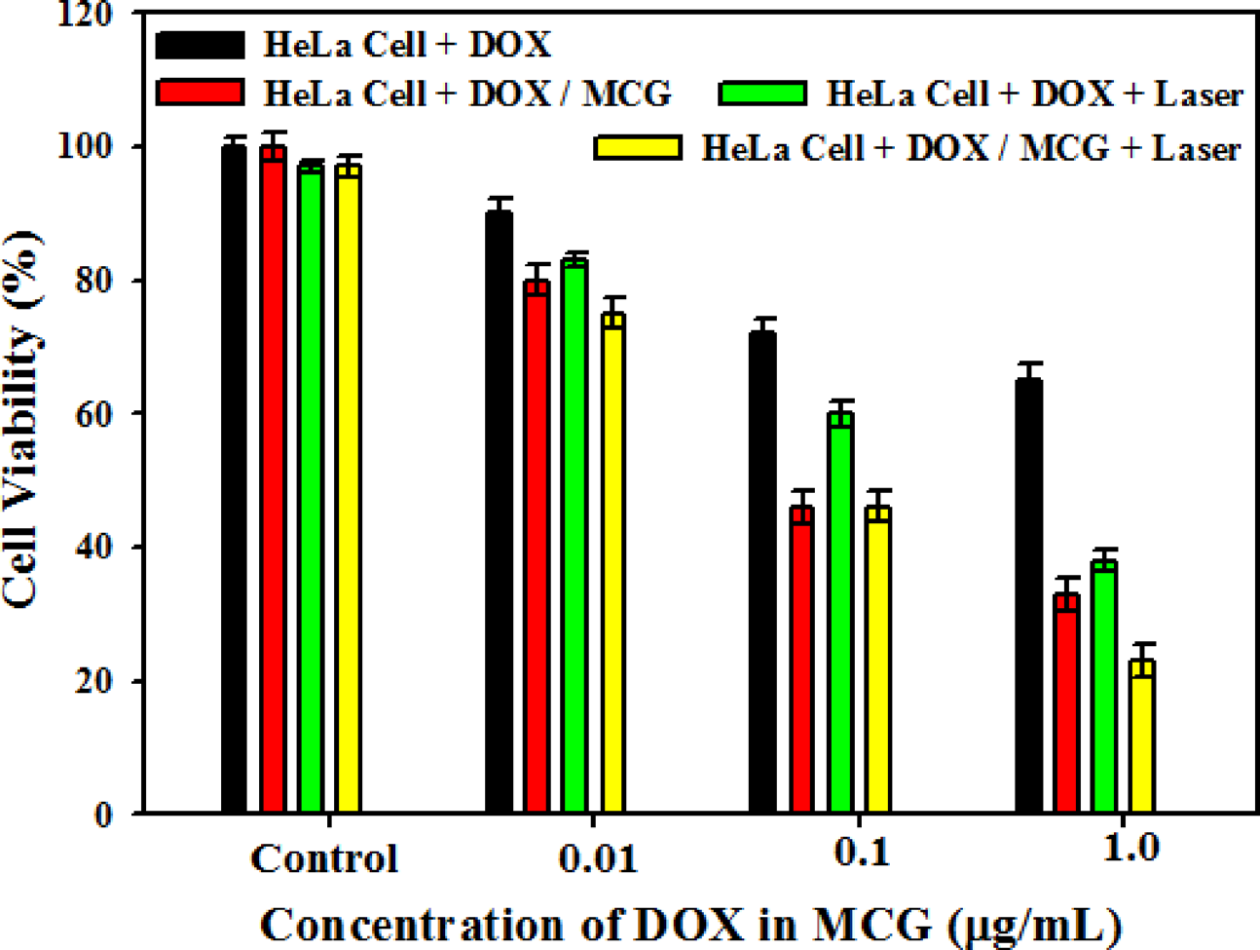
Cytotoxicity of Hela cells with MCG in different concentrations of DOX with and without laser irradiation.

The synergistic therapeutic efficacy of DOX/MCG against cancer cells were investigated by MTT assay after incubation with HeLa cells, cells treated with a NIR laser irradiation (808 nm, 1 W/cm^2^) after 10 min. As seen in Fig. 8, the cell viability of DOX treated cells was above 65% in all concentrations, showing drug has high biocompatibility. But, by NIR irradiation, the relative viability of Hela cell decrease very good.

This figure also illustrates that cell viabilities of DOX/MCG nanocomposite was lower than DOX at an equivalent dose of drug, indicating the chemotherapy effect of the MoS_2_ nanocomposites. On the other hand, the cell viability in DOX/MCG group under laser irradiation was lower than without laser irradiation, indicating the photothermal therapy effect of the MCG nanocomposites. Therefore, cell viability in the synergistic therapy group (DOX/MCG + laser) was much lower than DOX/MCG sample without laser irradiation. The result demonstrates that the MCG nanocomposites can kill solid tumors through synergistic therapy. It is worth noting that NIR laser irradiation both induces heat for photothermal therapy and accelerates the release of DOX from the nanocomposite through heat stimulated interactions between DOX and nanocomposite^40^. This leads to enhanced chemotherapy and the cytotoxic effect of the sample under laser irradiation was intensely enhanced in HeLa cells compared to without laser irradiation. All these results confirmed that the constructed multifunctional drug delivery system was effective in killing the tumor cells and is promising in chemo-photothermal combined cancer therapy.

## Conclusion

In the present study, a multifunctional MoS_2_-based drug delivery system (MCG nanocomposite) was successfully synthesized and shown to allow tumor-targeting synergistic chemo-photothermal therapy. The structural and optical properties for MCG nanocomposite was investigated via TEM, XRD, Zeta potential, DLS, FTIR, and UV-Vis spectroscopies to confirm that Fe_3_O_4_, CuS nanoparticles, GNRs have been incorporated non-uniform between MoS_2_ nanoflakes. The photothermal results of samples indicated that MCG nanocomposite produced higher photothermal heat than each individual sample alone (808 nm NIR laser irradiation at a power density of 1 W/cm^2^ after 10 min). The highest photothermal heat obtained from nanocomposite in 200 mg/mL concentration. Then, PEG was added nanocomposite to achieve excellent biocompatibility. The results confirm that the release of DOX in pH = 5.5 environments and under NIR light irradiation was faster than pH = 7.4, which is promising for tumor-specific delivery. So, increasing of nanocomposite concentration, pH, NIR irradiation and increasing of DOX concentration in sample are important factors in overcoming to cancerous cells. Therefore, we claim that, DOX/MCG nanocomposite under NIR light irradiation can open a new venue in cancer therapy process and efficiently can kill cancer cells and avoid destruction of surrounding healthy tissues.

## Data Availability

Data is provided within the manuscript file.
